# Multiple eccrine hidrocystomas on the bilateral forearms

**DOI:** 10.1016/j.jdcr.2026.06.003

**Published:** 2026-06-11

**Authors:** Kassidy Leibowitz, Candice E. Brem, Margaret S. Lee

**Affiliations:** a Boston University Aram V. Chobanian & Edward Avedisian School of Medicine, Boston, Massachusetts; b Section of Dermatopathology, Department of Dermatology, Boston University Aram V. Chobanian & Edward Avedisian School of Medicine, Boston, Massachusetts; c Department of Dermatology, Boston University Aram V. Chobanian & Edward Avedisian School of Medicine, Boston, Massachusetts

**Keywords:** adnexal neoplasm, dermal nodules, eccrine hidrocystoma, extra-facial presentation, multiple eccrine hidrocystomas

## Introduction

Eccrine hidrocystomas are benign, cystic skin lesions derived from eccrine sweat glands.^1^ They are generally divided into solitary (Smith type) and multiple (Robinson type) variants.^2^ Clinically, they characteristically present as translucent, flesh-colored to bluish papulonodular, dome-shaped skin lesions on the periorbital or malar facial regions. Involvement of extra-facial sites, including the chest, axilla, and neck, have been reported but remain uncommon.^3^

We report a case of multiple eccrine hidrocystomas presenting as dermal nodules on the bilateral volar forearms in a young patient, an unusual clinical distribution for this entity.

## Case report

A 21-year-old male presented with a 2-year history of progressively increasing numbers of small asymptomatic nodules on his bilateral forearms. He denied pain, pruritus, trauma, or overlying skin changes. There was no history of hyperhidrosis, excessive heat exposure, or changes in physical activity. He also denied prior history of inflammation in the affected areas, including eczema or dermatitis. His review of systems was negative. His medical history was only notable for a history of acne vulgaris of the face. Personal and family history were negative for ectodermal dysplasia, thyroid disease, or other relevant conditions.

On examination, no lesions were visible on immediate inspection of the bilateral volar forearms. However, when pulling the skin taut on the bilateral forearms, multiple nontender, round, dermal nodules became visible and palpable. More than a dozen lesions were appreciated, ranging from 1 mm to 4 mm in diameter (Fig 1).

**Fig 1:**
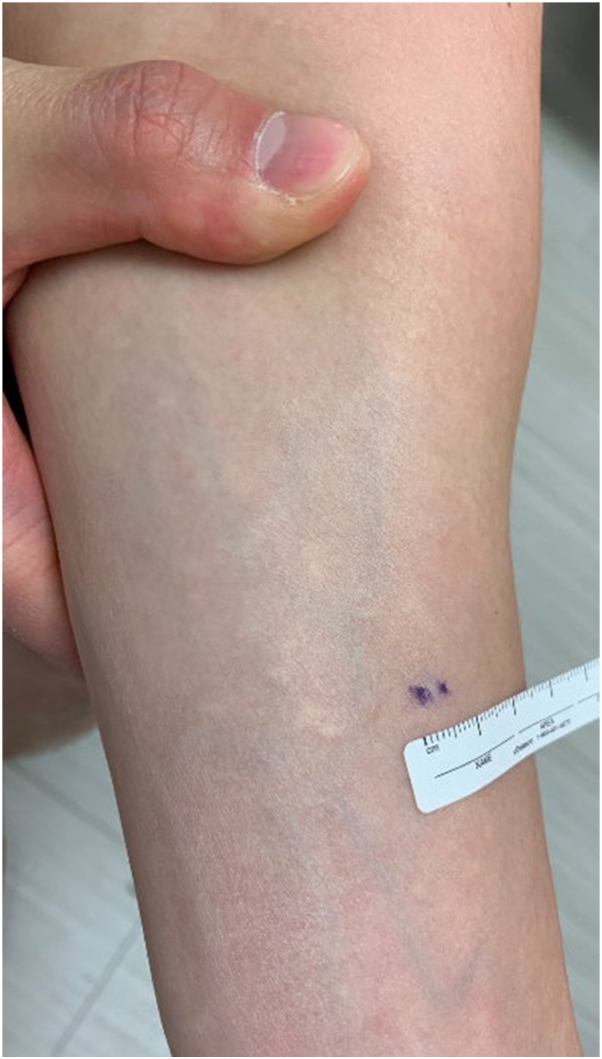
Clinical presentation of multiple eccrine hidrocystomas on the left volar forearm. The lesions appear as dome-shaped, flesh-colored papules visible only upon skin stretching. The biopsy site is indicated by purple ink.

The differential diagnosis included cystic lesions and adnexal neoplasms. Given the patient’s account of an increase in quantity and size of the lesions over the past 2 years, a biopsy was performed to establish a diagnosis.

As all lesions were clinically similar other than size, it was presumed that all the lesions were of the same entity. To minimize scarring, a single representative lesion on the left flexor forearm was selected for biopsy (Fig 1). A 4 mm punch biopsy of the lesion was obtained. Histopathologic examination revealed a unilocular cyst within the dermis lined by a double layer of cuboidal epithelial cells, consistent with an eccrine hidrocystoma (Fig 2, *A* and *B*).

**Fig 2:**
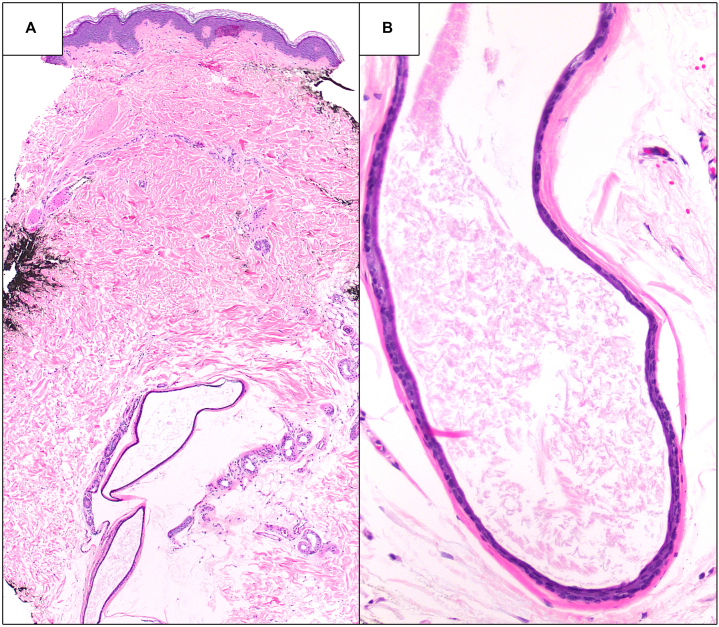
**A,** Low power examination (H&E, 20×) of the left forearm biopsy demonstrates a mid-to-deep dermal cystic structure. **B,** Higher power examination (H&E, 200×) reveals the cystic structure to be lined by a double layer of cuboidal epithelium consistent with an eccrine hidrocystoma. *H&E*, Hematoxylin and eosin stain.

Management options, including surgical excision, topical and systemic therapies, and intradermal botulinum toxin injections, were discussed. Given that the lesions were presumed to all be of the same benign entity and his biopsy scar was more noticeable than the lesions themselves, the patient declined further interventions, electing for observation.

## Discussion

This case describes an unusual presentation of eccrine hidrocystomas, with lesions on the bilateral volar forearms of a young, otherwise healthy male and a deeper dermal distribution than is typically reported.

Eccrine hidrocystomas are thought to arise from the obstruction of eccrine ducts with subsequent retention of sweat, leading to cystic dilation. Environmental and physiologic factors such as heat, humidity, and increased perspiration, have been implicated as contributing factors.^4^^,^^5^ In this patient, however, there were no identifiable triggers preceding the development and presentation of multiple eccrine hidrocystomas, suggesting that eccrine hidrocystomas may occasionally arise in the absence of identifiable external triggers.

The deeper location of lesions in this case is also noteworthy. Most eccrine hidrocystomas are identified histologically within the superficial-mid dermis, although deeper lesion involvement has been reported.^6^ This variation in dermal location may reflect differences in the level of eccrine ductal obstruction, with more proximal obstruction potentially involving deeper structures of the eccrine apparatus.

Solitary eccrine hidrocystomas occur with similar frequency in both sexes, whereas multiple eccrine hidrocystomas are more frequently reported in women, with some recent series suggesting a strong female predominance of up to 90% to 95%, although these findings are limited by small sample sizes.^7^ In contrast, other cohort studies describe a more modest sex predilection, though subtype-specific analyses distinguishing solitary lesions from multiple lesions by sex remain sparse.^8^ Overall, this case suggests that the demographic range of multiple eccrine hidrocystomas may be broader than traditionally appreciated.

Management of eccrine hidrocystomas is variable, particularly in cases involving numerous lesions. Surgical excision is effective for solitary eccrine hidrocystomas, with a recurrence rate as low as 4.7%, but it is impractical for the management of numerous lesions due to the risk of scarring.^9^ A variety of alternative therapies, including laser treatments, chemical ablation, anticholinergics, and botulinum toxin injections, have been described; however, comparative data on the efficacy of these treatments are limited.^5^

This case highlights an atypical presentation of eccrine hidrocystomas involving the volar forearms of a young male patient and emphasizes the importance of considering eccrine hidrocystomas in the differential diagnosis of subtle dermal nodules in atypical locations. Awareness of such presentations may help prevent misdiagnosis and unnecessary procedures. Further studies are needed to better characterize clinical variants and optimize management of multiple eccrine hidrocystomas.

## Conflicts of interest

None disclosed.
